# Supplementary material for: Polarization Angle Dependence of Optical Gain in a Hybrid Structure of Alexa-Flour 488/M13 Bacteriophage
Source: Nanomaterials (Basel). 2021 Dec 6;11(12):3309. doi: 10.3390/nano11123309 (PMC8707841; doi:10.3390/nano11123309)

Supporting information

S1. How to obtain stripe length dependent gain spectrum

Given the ASE spectrum for various stripe lengths $I(\hbar\omega, x)$, light amplification along a one-dimensional optical stripe can be described in terms of gain coefficient $g(\hbar\omega, x)$, the spontaneous emission density J_{spon} and solid angle of edge emission Ω.

$$\frac{\mathrm{d}I(\hbar\omega, x)}{\mathrm{d}x} = g(\hbar\omega, x)\ I(\hbar\omega, x) + J_{\mathrm{spon}}\Omega$$

$$g(\hbar\omega, x) = \frac{\overbrace{\frac{\mathrm{d}I(\hbar\omega,\ x)}{\mathrm{d}x}}^{\text{STEP-2}} - \overbrace{J_{\mathrm{spon}}\Omega}^{\text{STEP-3}}}{\underbrace{I(\hbar\omega, x)}_{\text{STEP-1}}}$$

As a first step, ASE intensity for stripe length $I(x)$ can be plotted for a selected spectral energy (or wavelength). If a gain coefficient is positive (g>0), the intensity shows a super-linear increase. On the other hand, the intensity increases linearly for g~0. In this case, the slope is the spontaneous emission term (JΩ).

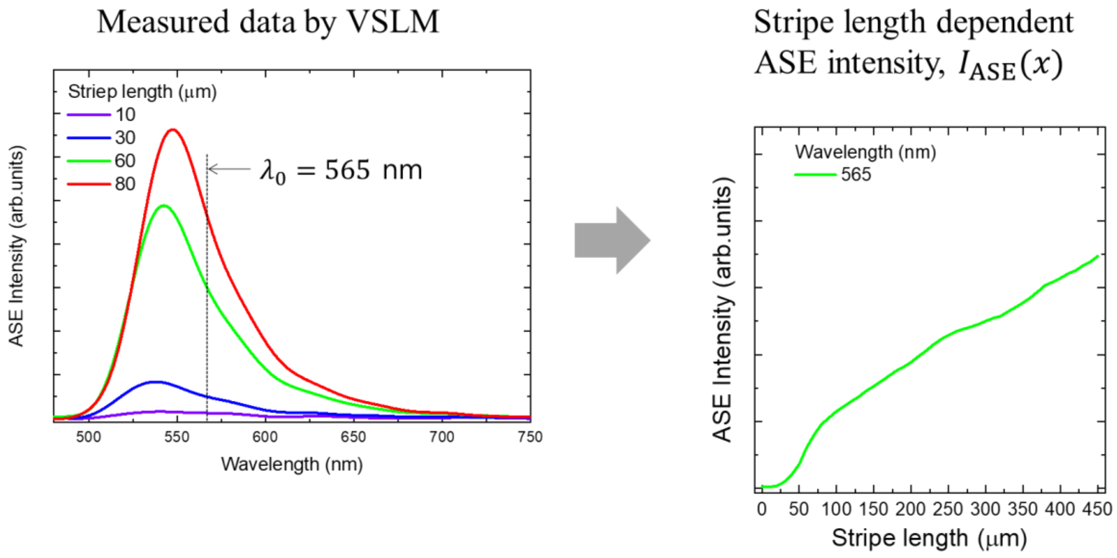

For a second step, we obtained derivative $\frac{\mathrm{dI}(x)}{\mathrm{d}\lambda}$ for stripe length, whereby the presence of gain can be noticed, and the linear slope near zero stripe length (JΩ) was also obtained. Finally, a stripe length dependence of the gain coefficient at a specific wavelength $g(\hbar\omega, x)$ can be obtained by $(\frac{\mathrm{dI}(x)}{\mathrm{d}x} - J_{spon}\Omega)/\mathrm{I}(x)$. To obtain a gain spectrum, this calculation process should be repeated for changing wavelength. As a result, this method enables to plot not only a stripe length dependence of gain coefficient at a certain wavelength but also a gain spectrum for different stripe lengths.

Stripe dependent gain calculation, $g(x)$

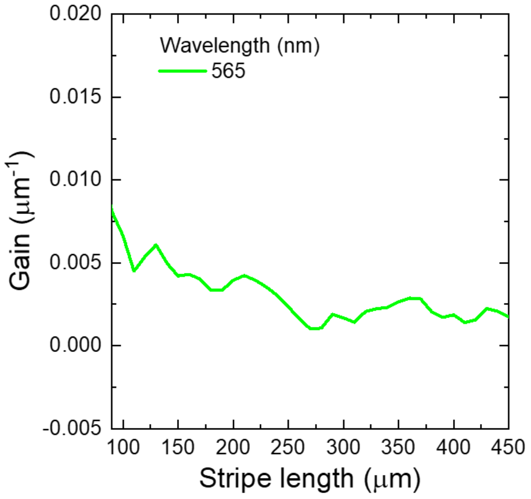

Wavelength dependent gain calculation, $g(\lambda)$

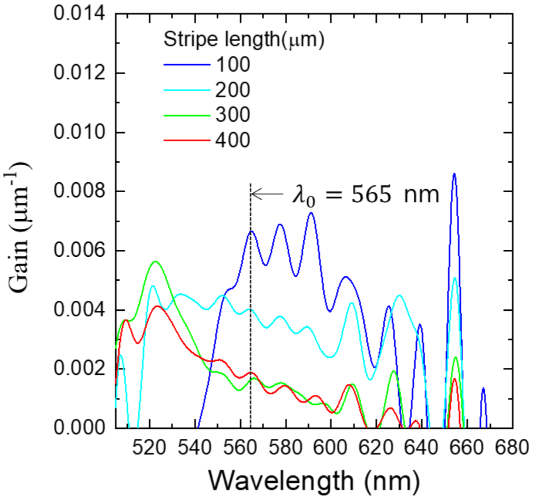

S2. Estimating an effective cavity length from etalon

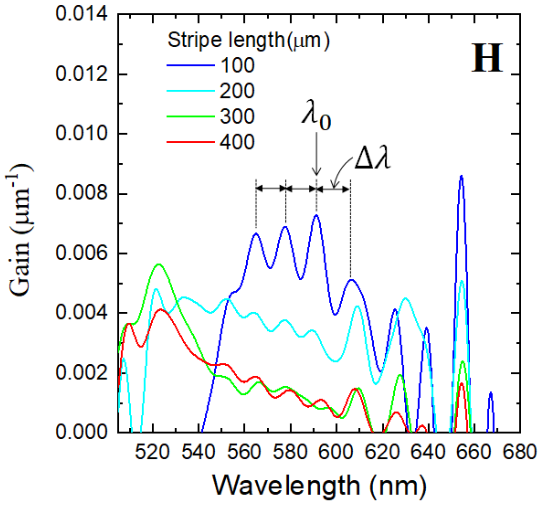

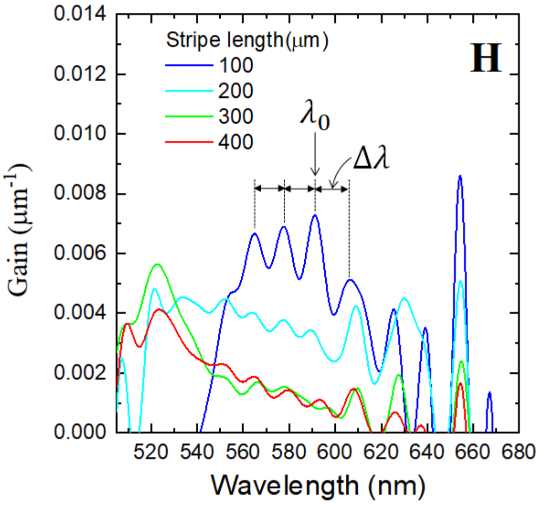

Given the mode spacing ($\Delta\lambda$), measured from the gain spectrum, the effective cavity length (L) can be estimated using equation-(5) as introduced in the main manuscript,

$$\Delta\lambda = \frac{\lambda_0^2}{2L(n - \lambda_0 dn/d\lambda)}$$

, where n is the refractive index at λ_0, and L is the cavity length.

To measure the refractive index of Alexa flour 488, we used the incidence angle dependence of interference fringes as shown schematically. For a selective monochromatic wavelength, the number of interference fringe change (N) was counted by changing the incident angle. Therefore, given the wavelength separation ($\Delta\lambda$), emission wavelength (λ_0), and refractive index spectrum, the cavity length (L) can be estimated. In the wavelength of our ASE spectrum, the refractive index can be considered a constant of n~1.34 with a dispersion $\frac{\mathrm{dn}}{\mathrm{d\lambda}}$~-9.5.

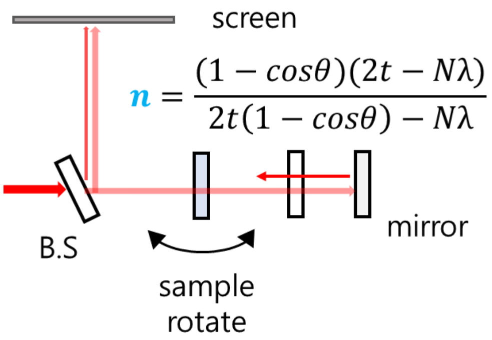

Supplement: Supplementary file 1 [file nanomaterials-11-03309-s001.zip › nanomaterials-1473819-supplementary.pdf]
